# Involvement of Lipids in the Pathogenesis of Amyotrophic Lateral Sclerosis

**DOI:** 10.3390/life13020510

**Published:** 2023-02-12

**Authors:** Alisa V. Alessenko, Uliana A. Gutner, Maria A. Shupik

**Affiliations:** Emanuel Institute of Biochemical Physics, Russian Academy of Sciences, Kosygin Str. 4, 199334 Moscow, Russia

**Keywords:** amyotrophic lateral sclerosis (ALS), models of ALS, lipids, sphingolipids, enzymes of sphingolipids metabolism

## Abstract

Amyotrophic lateral sclerosis (ALS) is a fatal neurodegenerative disease characterized by the progressive degeneration of upper and lower motor neurons. To study its underlying mechanisms, a variety of models are currently used at the cellular level and in animals with mutations in multiple ALS associated genes, including SOD1, C9ORF72, TDP-43, and FUS. Key mechanisms involved in the disease include excitotoxicity, oxidative stress, mitochondrial dysfunction, neuroinflammatory, and immune reactions. In addition, significant metabolism alterations of various lipids classes, including phospholipids, fatty acids, sphingolipids, and others have been increasingly recognized. Recently, the mechanisms of programmed cell death (apoptosis), which may be responsible for the degeneration of motor neurons observed in the disease, have been intensively studied. In this context, sphingolipids, which are the most important sources of secondary messengers transmitting signals for cell proliferation, differentiation, and apoptosis, are gaining increasing attention in the context of ALS pathogenesis given their role in the development of neuroinflammatory and immune responses. This review describes changes in lipids content and activity of enzymes involved in their metabolism in ALS, both summarizing current evidence from animal models and clinical studies and discussing the potential of new drugs among modulators of lipid metabolism enzymes.

## 1. Introduction

Amyotrophic lateral sclerosis (ALS) is a fatal neurodegenerative disease characterized by the progressive degeneration of motor neurons in the motor cortex, brainstem, and spinal cord. Clinical symptoms include fasciculations, progressive muscular weakness and atrophy, impaired speech and swallowing, and increased reflexes. The main cause of death, which typically occurs on average three to five years after disease onset, is respiratory failure [1]. About 90% of all reported ALS cases have an unknown etiology and are classified as sporadic. The remaining 10% are hereditary disease forms characterized by mutations in a number of genes, mostly of an autosomal dominant nature [2]. Key mechanisms involved in the disease include excitotoxicity [3], oxidative stress [4], mitochondrial dysfunction [5], neuroinflammatory responses, and immune responses [6]. Recently, the mechanisms of programmed cell death (apoptosis), which may be responsible for the degeneration of motor neurons observed in the disease, have been intensively studied [7,8,9]. The extreme genetic variability associated with ALS contributes to explain the complexity of the disease, in which heterogeneous mechanisms lead to a common pathogenesis. In this regard, numerous studies have focused on the role of lipid metabolism changes in ALS pathogenesis.

Lipids play an extremely important role in the central nervous system (CNS). They perform a structural function as the main components of cell membranes, serve as a source of energy, and participate in intercellular communication and transmission of cell signals. The importance of lipids in biological systems is emphasized by the fact that 5% of human genes are responsible for lipid synthesis [10]. All classes of lipids are widely represented in the brain [11], and, with the exception of adipose tissue, the brain is the organ with the highest lipid content in humans and animals [12]. Impaired lipid balance has a negative effect on structural and physiological properties of the brain, as well as the functions of neurons and neuroglia, including membrane transport and enzyme activity control. Membrane lipid dysregulation leads to impaired neurotrophic functions and apoptosis of neurons in neurodegenerative diseases [13]. Destruction of the cell membrane is a characteristic feature of neurodegeneration that occurs in chronic diseases of the CNS. Various disorders of lipid metabolism are typical of both sporadic and hereditary forms of ALS. The most frequently observed among these are dys- or hyperlipidemia and oxidative stress [14,15,16]. One of the characteristic signs of ALS, observed in approximately two thirds of patients, is weight loss due to hypermetabolism, which leads to increased energy consumption of the body and, since lipids are one of the main energy sources, this phenomenon is directly related to lipid metabolism [15].

Among lipids, a key role is played by sphingolipids, which are the most important sources of second messengers transmitting signals of cell proliferation, differentiation, and apoptosis [17]. Defects in sphingolipid metabolism have also been found in hereditary metabolic diseases, such as Fabry and Niemann–Pick, in lysosomal storage disorders, such as Gaucher disease, and in neurodegenerative pathologies, such as Parkinson’s and Alzheimer’s diseases [10,14,18,19,20]. Recently, special attention has been paid to the study of the involvement of sphingolipids in the pathogenesis of ALS due to the multiplicity of their functions in the structure and physiology of the brain. Sphingolipids can play a special role in the induction of apoptosis, which is characteristic of this disease [11,12].

The regulatory role of lipids as second messengers is further revealed by the study of inflammatory processes in ALS, which are accompanied by microglia activation, loss of neuromuscular junctions, and subsequent degeneration of motor neurons. At the same time, there is an increased content of neurotoxic molecules, such as cytokines, which have a negative effect on the processes of protein and lipid synthesis, where second messengers of lipid nature are actively involved.

Thus, the study of lipids in ALS can offer new insights into the mechanisms responsible for the processes of neurodegeneration, inflammation, oxidative stress, cell signal transmission, changes in the structure of membranes, and also influence the development of new methods for diagnostics and targeted therapy for ALS.

## 2. Animal Models Used for Studying ALS Mechanisms

To study the mechanisms of any disease, particularly ALS, it is necessary to have its models both at the cellular level and in animals. The first breakthrough in the development of models for the study of ALS using rodents was the discovery, in the early 1990s, that mutations in the gene encoding superoxide dismutase 1 (SOD1) were associated with this disease [21]. The vast majority of familial cases are indeed caused by genetic mutations in C9ORF72 (about 40–50%) SOD1 (15%) genes [21]. To date, more than 160 mutations in the SOD1 gene have been found to be associated with the pathogenesis of ALS. [22]. Based on mutations in the SOD1 gene, transgenic animal models have been created, which currently represent an elective tool for studying the molecular mechanisms of ALS pathogenesis [1,21,22,23].

The discovery of mutations in the SOD1 gene in ALS has led to studies on the role of free radicals and oxidation processes, proteins, DNA, and lipids (including membrane phospholipids) in the pathogenesis of the disease [21,23,24]. However, it has been later found that mutations in the SOD1 gene are not the only ones associated with oxidative processes in ALS, since these mechanisms were also observed in cases carrying mutations in other ALS associated genes [6]. Nevertheless, since an increased content of products of lipid peroxidation (LPO) occurring in the CNS has been found in the cerebrospinal fluid, plasma, and urine of patients with ALS, it has been lately proposed to search for disease biomarkers among the products of oxidative stress [25,26].

### 2.1. Models with Expression of Mutant SOD1 Isoforms

Models of ALS have been created in various rodent species, including mice and rats, in which mutant SOD1 isoforms have been expressed. Mouse models overexpressing mutant forms of human SOD1 have become the most popular. Mouse models have also been developed, expressing multiple copies of the SOD1 mutant genes with early fatal motor neuron disease [27]. Animals expressing altered SOD1 develop a motor neuron disease resembling human disease, with different periods of manifestation and rates of progression. The core features of the disease in these animals are early stro- and microgliosis, glutamate-mediated excitotoxicity, deficiency of axon transport, vacuolization of mitochondria, disruption of the structure of neurofilaments, and reduced metabolic support of motor neurons by surrounding glial cells [27,28,29]. The result is a selective loss of spinal motor neurons, causing widespread muscular weakness and atrophy in both the hind and forelimbs, leading to paralysis and death. An important difference between these mouse models and human disease features is that significant cortical neuronal degeneration, which is a fundamental characteristic of human disease, is not observed in most of these models [29]. Notably, this critical difference may represent the major pathogenic shortcomings of the SOD1 mouse model as a tool for studying motor neuron diseases [30].

However, over the past 20 years, mouse models of ALS expressing SOD1, mainly the SOD1^G93A^ variant and further the SOD1^G37R^, SOD1^G85R^, and SOD1^G86R^ ones, have been used to characterize the pathology of ALS, as well as to study the specific benefits of potential treatments [30,31]. The popularity of such models have been determined by the presence of demyelinated axons, as well as by the fact that the expression levels of MCT1 transporters in mice have been found to be reduced as observed in the cortex of ALS patients [32]. In addition to murine models, transgenic rat strains overexpressing SOD1 have been developed, which have proven to be particularly useful for evaluating therapeutic studies due to the animal size, especially when administering therapeutic drugs, such as continuous intraspinal delivery of therapeutic compounds through osmotic mini pumps [31,33,34].

### 2.2. Models with Mutations in Proteins Involved in RNA Metabolism

Multiple studies have shown that, in most ALS patients, the pathology is caused by disturbances in RNA metabolism, which is the main difference from animal models with mutations in the SOD1 gene [35,36,37,38,39,40,41,42,43]. No mutations in the SOD1 gene have been detected in patients with ALS and frontotemporal dementia, however, those cases presented TDP-43-positive cytoplasmic inclusions in motor neurons [35,36], and this observation has led to a closer focus on the role of altered RNA metabolism in the pathogenesis of ALS. The identified protein aggregates were ubiquitinated, phosphorylated, and contained cleaved C-terminal fragments of TDP-43-transactive response DNA binding protein 43 kDa. The hypothesis that ALS is an RNA proteinopathy has been further confirmed by the identification of mutations in the TAR DNA-binding protein (TARDBP) gene (coding for TDP-43), which occur in approximately 3% of patients with familial ALS [37,38]. Given the role of TDP-43 in RNA processing, transport, and splicing, this observation has led to the hypothesis that altered RNA metabolism might be involved in the pathogenesis of the disease [39,40]. In addition to TARDBP, mutations in the gene coding for another DNA/RNA-binding protein, fused in sarcoma (FUS), have also been found in ALS. FUS and TDP-43 have a similar domain structure and perform similar functions in the cell: they are involved in mRNA regulation, processing, and transport [40,41,42,43]. In addition to nucleic acid binding, FUS is further involved in both general and specialized functions, including transcription initiation. FUS interacts with several nuclear receptors and the gene-specific transcription factors Spi-1/PU.1 and NF-κB. It can also influence transcription initiation and promoter selection by interacting with RNA polymerase II and the TFIID complex [44,45,46]. Additionally, unlike TDP-43, FUS aggregates have been found only in patients carrying FUS mutations and not in other familial or sporadic ALS cases, suggesting that, unlike TDP-43, the contribution of FUS to the disease may be limited to a small subset of patients. Nevertheless, ALS animal models with mutations in genes coding for this protein, the so-called FUS proteinopathies, have been increasingly proposed [47,48].

Notably, however, given that the majority of familial ALS cases seem to be determined by changes in RNA metabolism [47], more attention has been recently paid to the development of new models, such as models of transgenic rodents with C9orf72 mutations [48,49].

### 2.3. Models with Mutations in Newly Discovered Genes

Over the past 10 years, there has been a surge in the identification of genes associated with ALS. Some of them, such as UBQLN2, SOSTM, VCP, PFN1 and MATR3, have been rarely seen in hereditary phenotypes, but they conversely seem to be involved in the pathogenesis of sporadic disease forms, as they are often present in the CNS tissues of these patients [50]. Mutations in VCP have been suggested to be involved in the pathogenesis of ALS, frontotemporal dementia, and Paget’s disease [51]. The newly obtained rodent models expressing mutant VCP [52,53] exhibit the symptoms of Paget’s disease accompanied by progressive muscular weakness and significant accumulation of cytoplasmic TDP-43 in neurons of the brain and in spinal cord motor neurons. Combining both new rodent models of ALS and iPSCs with patient-specific features in research will lead to a better understanding of ALS pathogenesis and guide the identification of new potential therapeutic targets.

## 3. Hyperlipidemia in ALS

Numerous studies are currently underway to search for lipid biomarkers of ALS. The plasma lipid profile of ALS patients (triglycerides, cholesterol, low-density lipoprotein (LDL) and high-density lipoprotein (HDL) concentrations) has been suggested as a potential marker of disease severity and life expectancy in patients with ALS. [16,54,55,56].

**Figure 1:**
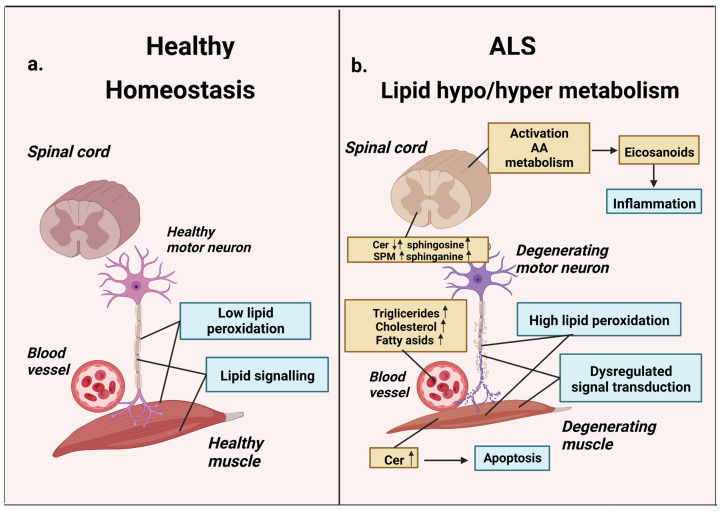
Lipid metabolism in homeostasis and ALS. (**a**) Normal lipid metabolism: low peroxidation, normal lipid signaling, normal motor neuron, and muscle functions. (**b**) ALS-related lipid dysfunction: hypo/hyper lipidemia, high peroxidation, lipid-signaling dysregulation. Blood plasma cholesterol, triglycerides, and fatty acids are elevated in ALS disease states. Activation of AA-metabolism causes synthesis of arachidonic acid-derived eicosanoid classes and associated inflammatory effects. ALS-related changes in SPM/ceramide signaling lead to the accumulation of proapoptotic ceramides and sphingosine. These agents can induce apoptosis, causing degeneration of motor neurons and muscles (AA—arachidonic acid, SPM sphingomyelin, Cer—ceramide). Up arrow icon means that the content of the metabolite is increased, the down arrow icon means that it is reduced. Up and down arrows mean that date are inconsistent.

Lipid disbalance in the blood of ALS patients manifests itself in the form of both dyslipidemia and hyperlipidemia (Figure 1, Table 1) [15]. Moreover, as it is shown in many studies, the development of hyperlipidemia in ALS patients correlates with an increase in their life expectancy. For example, in the work by L. Dupuis et al. [16], a twofold increase in the content of total lipids in the blood of ALS patients correlated with a 12 months increase in life expectancy. Moreover, recent studies have shown that elevated serum triglycerides correlate with longer life expectancy in ALS [54].

However, there is increasing evidence that in ALS patients most of these measures do not significantly differ from healthy controls and may not represent reliable prognostic markers in ALS. In studies with large sample sizes, no significant differences have been found in the lipid profile of blood serum in terms of cholesterol, triglycerides, LDL and HDL [56]. In addition, no association has been observed between lipid profile and life expectancy [54].

### 3.1. Triglycerides

One of the factors thought to contribute to neuronal survival in ALS is elevated triglyceride content in blood plasma, and a recent study evaluating 413 ALS patients and 400 controls has shown that patients with elevated triglyceride content exhibit a longer life expectancy [54]. Other studies have further found a significant gender effect, with higher triglyceride levels in a specific HDL lipoprotein fraction correlating with longer survival in women, but not in men [61]. Without conditionality, as many researchers have noted, triglyceride content largely reflects a patient’s nutritional level, which in turn may influence survival in ALS [57]. The cerebrospinal fluid (CSF) of ALS patients has also been shown to contain low levels of long-chain triglycerides (16:1/18:1/18:2) [57].

### 3.2. Phospholipids

Phospholipids play a critical role in metabolism of nerve cell systems; they are major structural components of outer and inner cell membranes, affecting membrane fluidity and the activity of membrane-bound proteins, and are also involved in cell signaling as a source of secondary messengers. The development of lipidomics in recent decades has been primarily associated with studies on the role of phospholipids in normal brain function and in various diseases, including neurodegenerative ones. Phospholipids have been shown to be involved in key processes in Alzheimer’s disease, Parkinson’s disease, and multiple sclerosis [11,17]. However, there are extremely few studies focusing on the function of phospholipids in ALS pathology. Works investigating the phospholipid profile of CSF in ALS patients and animal models have shown that the levels of certain forms of phospholipids with long-chain fatty acid residues specifically differ from controls, phosphatidylcholine (20:4), and sphingomyelin (22:0), in particular [11]. Similar results have been observed in transgenic mice brains, with significantly higher phosphatidylcholine (36:2), phosphatidylcholine (36:4), and phosphatidylcholine (40:6) observed compared to controls. The difference in phosphatidylcholine content between ALS patients and controls, as well as between the transgenic ALS model and controls, suggests a significant involvement of phosphatidylcholine, especially phosphatidylcholine (36:4), in ALS pathology.

Recent studies suggest that increased concentration of phosphatidylcholines, especially phosphatidylcholine (16:0/20:4), may induce increased phospholipase A2 activity, resulting in augmented release of lipid mediators such as eicosanoids, leading to stimulation of inflammation in ALS [11] Similarly, it has been suggested that the release of arachidonic acid may lead to the subsequent synthesis of prostaglandins and lipid peroxidation, which is typical of neurodegenerative processes [62]. Similarly, it was found that the concentration of phospholipids containing the 22:6 fatty acid chain was increased in the mouse model of ALS, which may correlate with docosahexaenoic acid involved in the processes of neuronal membrane stabilization, signal transduction, neuronal differentiation, neurogenesis, and others. While in ALS mice models some works have shown an association between motor neurons loss in the spinal cord and a reduction in phosphatidylcholine (di- acyl-16:0/22:6), other studies have reported increased content of docosahexaenoic acid in the frontal cortex of ALS patients [11,58].

Another work has investigated the content of phospholipids in the SOD model. Different parts of the spinal cord of transgenic SOD mice were studied at different stages of the pathology: at presymptomatic, symptomatic, and postmortem stages. These studies have shown a significant decrease in phosphatidylcholine containing docosahexaenoic acid: phosphatidylcholine (diacyl-16:0/22:6), phosphatidylcholine (diacyl-18:0/22:6), and phosphatidylcholine (18:1/22:6) during the terminal life stage of animals, which was accompanied by neuronal loss. The content of other molecular species of phosphatidylcholine (diacyl-16:0/16:0) did not actually differ from controls. Researchers concluded that, in the terminal stages of the pathology, the decrease in docosahexaenoic acid-containing phosphatidylcholines, but not other phosphatidylcholines, might reflect the loss of anterior horn neurons of the spinal cord [58].

### 3.3. Polyunsaturated Fatty Acids

Fatty acids, free or included in triglycerides and phospholipids, are a source of energy and a substrate for lipid peroxidation, being destroyed with the formation of hydrophobic radicals when interacting with reactive oxygen species. Notably, increasing attention has been paid to the study of polyunsaturated fatty acids (PUFAs), which, as structural components of phospholipids, affect the permeability and fluidity of membranes, as well as the activity of membrane-bound enzymes and the transport of proteins. In addition, PUFAs are a substrate for the synthesis of pro-inflammatory mediators involved in the pathogenesis of ALS, such as the eicosanoids (prostaglandins, prostacyclins, thromboxanes, and leukotrienes). Some works have suggested that the fatty acid composition of total lipids in patients’ blood may reflect the pathological condition in ALS. Palmitoleate (16:1) and oleate (18:1) have been shown to be significantly elevated compared to controls, and a ratio of 16:1/16:0 has been found to correlate with ALS Functional Scale (ALSFRS-R) scores. Therefore, the 16:1/16:0 ratio has been lately proposed as an independent prognostic factor in ALS [59].

Long-chain PUFAs play a key role in brain cells. They regulate the interaction between neural and glial cells, stimulate synaptogenesis, and interact with neurotransmitters. The biological role of PUFAs in the brain depends on both the length of the carbon chain and the location of the double bonds [63].

Linoleic and alpha-linolenic acids are considered essential and enter the body through food. By penetrating the blood–brain barrier (BBB), alpha-linolenic acid can decrease the rate of lipid peroxidation by binding to the transcription factor NF-κB, preventing glutamate-related excitotoxic damage and oxidative stress, thereby preventing neuronal death. The neuroprotective properties of alpha-linolenic acid are also manifested in the prolongation of neuronal survival through the reduction in the immunoreactivity of pro-apoptotic proteins. In addition, alpha-linolenic acid serves as a metabolic precursor of eicosopentanoic and dexagexagenic acids. Furthermore, in ALS, alpha-linolenic acid has also been shown to have a positive effect, reducing the risk of developing the disease [63].

Docosahexaenoic acid (omega-3 PUFAs) is one of the key lipids involved in nervous system homeostasis. Sporadic ALS has been suggested to specifically alter the synthesis of docosahexaenoic acid, and excitotoxicity combined with oxidative stress leads to increased levels of docosahexaenoic acid and increased expression of enzymes of its synthesis. It has also been shown that docosahexaenoic acid metabolism is associated with TDP-43 protein aggregation, one of the main pathogenetic factors in ALS [64]. In addition, docosahexaenoic acid serves as a precursor of the neuroprotective agent resolvin D1, which has been proposed as a potential treatment in ALS. The mechanism of action of resolvin D1 is based on its role as a mediator in inhibiting the synthesis of pro-inflammatory cytokines IL-6 and TNF-α [65].

Another mediator of lipid nature, arachidonic acid, plays an important role in ALS. Arachidonic acid, which can be metabolized either by the enzyme prostaglandin-endoperoxy-N-synthase or lipooxigenase, depending on which becomes a precursor of pro- or anti-inflammatory components of the immune system, such as prostaglandins, leukotrienes, etc. Activation of arachidonic cycle enzymes, possibly reflecting the increased content of prostaglandins, has been observed in various neurodegenerative diseases, including ALS (in the spinal cord of model animals and in the cerebrospinal fluid of patients with ALS) [66,67]. Apparently, an increased intensity of the arachidonic acid metabolism leads to a decrease in its content in ALS, a result observed in cellular and animal models [68]. Arachidonic acid metabolism in ALS seems to be influenced by gender: high levels of arachidonic acid in women have been found to be associated with an increased risk of developing the disease [69].

Eicosopentaenoic acid, which metabolism is related to arachidonic acid, serves as a source of eicosanoids. Among omega-3 fatty acids, eicosopentaenoic acid has a neuroprotective effect. In ALS, an important role of eicosopentaenoic acid has been shown in the presymptomatic stage of SOD1-transgenic mice, leading to a significant reduction in microglia and astrocyte activation [70].

In addition to these well known polyunsaturated fatty acids and their functions, new PUFA derivatives have been recently discovered, such as compounds of polyunsaturated fatty acids with nitric oxide (nitroalkenes, NO_2_-fatty acids), which possess neuroprotective functions. NO_2_-fatty acids have been found in blood plasma, cell membranes, and tissues. They can affect inflammatory process by reducing the level of pro-inflammatory mediators by inhibiting enzymes, which have a protective effect and prevent the death of motor neurons in ALS.

NO_2_-fatty acids, as secondary messengers of intracellular signaling, trigger the signaling cascade through covalent reversible post-transcriptional modifications of nucleophilic amino acids, thereby affecting the regulation of protein and enzyme transcriptional processes. The function of intracellular signal transduction performed by NO_2_-fatty acids depends on their distribution and arrangement in hydrophilic and hydrophobic environments, esterification, and other properties. NO_2_-arachidonic and NO_2_-oleic acids are included in the activation signaling of the transcription factor Nrf2, the effect of which decreases the toxicity of activated astrocytes to motor neurons [71]. Currently, one of the most studied nitroalkenes is nitro-oleic acid, and its anti-inflammatory properties have been shown to be based on the ability to penetrate through the BBB and include a reduction in prostaglandin and hydroxyeicosatrienoic acid levels in the brain, as well as a reduction in astrogliosis in the spinal cord of ALS animal models (SOD1 mice) [72,73]. Notably, the attempts to use changes in the composition of PUFAs as specific markers for early diagnosis of ALS have not been successful: no correlation has been found between the content of most individual PUFAs and the risk of developing the disease [69].

### 3.4. PUFAs, Diet and Nutrition

Most PUFAs can be synthesized in animal and human cells, but many of them come from food. PUFA deficiency leads to a variety of disorders, from changes in the fatty acid composition of nerve cell membranes to neurological and cognitive deficiencies. On the other hand, it has been shown that PUFAs coming from food can penetrate through the BBB, enter brain cell membranes, and modulate oxidative stress and inflammatory processes (including the release of cytokines), mechanisms typically involved in ALS. There have been studies examining the effects of increased PUFAs in the diet of ALS patients and in animal models. Group long-term follow-up studies with large sample sizes (1,002,082 people) have shown that the inclusion of PUFAs in the diet, and in particular alpha-linolenic acid, significantly reduce the risk of developing ALS [63,74,75].

The results of a study on the effect of different diets on the course of ALS have highlighted a positive effect when a diet rich in ketones (D-β-3 hydroxy-butyrate) was used. D-β-3 hydroxybutyrate, penetrating through the BBB, is metabolized in brain mitochondria to acetoacetate and then to acetyl-CoA, thus serving as an energy substrate for nerve cell metabolism. The keto-new diet, already known for its neuroprotective properties in some neurodegenerative diseases (such as Parkinson’s disease), as well as in epilepsy, improves motor functions and survival of transgenic SOD mice. One modification of the ketone diet, a diet containing medium-chain (6 to 12 carbohydrates) triglycerides, has been further shown to contribute to a significant reduction in the progression of weakness and an increase in the lifespan in ALS-model animals [76].

## 4. Sphingolipids in ALS

The CNS contains a large amount of sphingolipids, the metabolites of which not only play a structural role in membranes, but are also sources of secondary messengers that carry out the transmission of numerous cellular signals. Sphingolipid structure is based on the aliphatic amino alcohol sphingosine. Sphingolipids are highly active biological compounds involved in the regulation of cell proliferation, differentiation, intercellular interactions, cell migration, extracellular and intracellular signaling, and cell death [17,77]. Sphingolipid metabolism disorders play an important role in the pathogenesis of neurodegenerative diseases, including Huntington’s chorea [78], Alzheimer’s disease [18,79], Parkinsonism, and others. [12,80]. Changes in sphingolipid metabolism can be observed in both sporadic and familial ALS and affect the rate of disease progression (Table 2). These changes involve both the total content of sphingolipid subclasses (ceramides, sphingomyelins, glycosphingolipids), as well as their specific molecular species [18,81,82].

### 4.1. Ceramides

Ceramide, composed of sphingosine and fatty acid, plays a key role in the metabolism of sphingolipids [83,84] and is a hydrophobic base for sphingolipids of other subclasses, including sphingomyelin, glucosylceramides, galactosylceramides, gangliosides, sulfatides, and others. In mammals, ceramide is part of hundreds of different molecules, and its metabolism is controlled by several dozens of enzymes [85,86]. A number of neuromuscular diseases are caused by mutations in genes that regulate ceramide metabolism. Type I hereditary sensory neuropathy is associated with mutations in SPTLC1, subunit 1 of the long chain serine palmitoyltransferase (SPT) (an enzyme that limits the rate of de novo synthesis of ceramide), which leads to an increase in SPT activity, ceramide content, and degeneration of motor neurons [87]. Mutations of the gene encoding 3-ketodihydrosphingosine reductase (FVT1), which catalyzes the second step of de novo synthesis of ceramide, lead to spinal muscular atrophy (SMA) in cattle [88]. Mutations in the acid ceramidase (ASAH1) gene (an enzyme of lysosomal ceramide hydrolysis) determine a whole range of motor neuron diseases: from Farber’s disease, a fatal disease of early childhood characterized by a decrease in ASAH1 activity by more than 90%, to atypical forms of SMA with a decrease in ASAH1 activity by 70% [89]. Therefore, any changes in ceramide levels may be a critical determinant of motor neuron survival [81]. Recently, new SPTLC1 mutations leading to sphinganine and increased ceramide synthesis have been identified and found to be associated with monogenic ALS disease forms in childhood [90].

**Table 2:** Key components of the sphingomyelin cycle in the spinal cord of model animals depending on the model and stage of ALS development; nd—no significant differences model animals and controls, ne—a parameter in a study has not been evaluated. Up arrow icon means that the content of the metabolite is increased, the down arrow icon means that it is reduced.

	Pre-Sympt	Early Sympt	Sympt	Model	References
Ceramide–sphingosine axis					
Ceramide	↑	ne	↑	mouse SOD1^G93A^	[91]
	ne	↓	↑	mouse SOD1^G93A^	[81]
	↓↑	ne	↓↑	mouse SOD1^G86R^	[92]
	ne	ne	↑	rat SOD1^G93A^	[93]
	↓	nd	nd	mouse FUS (1-359)	[94]
Asah1 mRNA expression	ne	↑	↑	mouse SOD1^G93A^	[95]
	nd	nd	↑	mouse FUS (1-359)	[94]
Sphingosine	nd	nd	↑	mouse FUS(1-359)	[96]
Complex sphingolipids, ceramide sources					
Sphingomyelin	nd	ne	↑	mouse SOD1^G93A^	[91]
	ne	↓	↓	mouse SOD1^G93A^	[81]
	↑↓	ne	↑	mouse SOD1^G86R^	[92]
Galactosyl ceramide	ne	↓	↓	mouse SOD1^G93A^	[81]
Glucosyl ceramide	ne	nd	↑	mouse SOD1^G93A^	[81]
	↓	ne	↓↑	mouse SOD1^G86R^	[92]
Gangliosides GM3	ne	↑	↑	mouse SOD1^G93A^	[81]
Gangliosides GM1	ne	↓	↓	mouse SOD1^G93A^	[81]

In the pioneering work by R. Cutler et al. [91], in patients with sporadic ALS and transgenic mice of the SOD1^G93A^ line, a model of the familial form of ALS, an increase in the content of two molecular species of ceramide (C16:0 and C24:0), and sphingomyelin C16:0 in the lumbar spinal cord have been observed. Moreover, the accumulation of ceramide C16:0 in animals was observed even at the presymptomatic stage of ALS. These changes did not affect the cervical spinal cord of SOD1^G93A^ mice during the studied periods, highlighting the vulnerability of lumbar neurons. The authors also showed that oxidative stress, which is an early event in the development of ALS, might determine the increase in the content of sphingolipids, and ceramide, which in turn, can directly affect mitochondria and enhance oxidative processes in cells [91]. A subsequent study by J. Dodge et al. [81], examining samples of gray and white matter of the cervical spinal cord in patients with sporadic ALS, has shown a significant increase in the total content of ceramide and its molecular species, C18:0, C24:1, and C24:0-OH. The increase in the content of ceramide was not associated with the decrease in the activity of enzymes mediating its degradation (which is typical of a group of diseases with impaired lysosomal metabolism). The activity of enzymes responsible for the formation of ceramide from hexosylceramides, glucocerebrosidases 1 and 2 (GBA1 and GBA2), and galactosylceramidase (GALC) increased at acidic pH values, and the activity of GBA2 and GALC increased at neutral pH values, indicating the possibility of intensifying the hydrolysis of sphingolipids in lysosomes, in the plasma membrane of the cell, and in organelles in which sphingolipids were synthesized [81]. In motor neurons of ALS patients, a decrease in the expression of subunit 2 of the long chain SPT has also been observed [97], suggesting the formation of ceramide predominantly through catabolic pathways, and not as a result of de novo synthesis [81].

Motor neuron apoptotic death in SOD1^G93A^ mice has been found to be accompanied by ceramide generation and activation of the enzyme neutral sphingomyelinase, which hydrolyzes sphingomyelin to ceramide [98]. Changes in the content of ceramide and the sphingolipids in the lumbar spinal cord of SOD1^G93A^ mice fundamentally depend on the stage of ALS. In a work by Dodge and colleagues [81], at the terminal stage of the disease, a slight increase in the level of C24:0-OH ceramide compared to controls has been found. However, in the earlier stages of the disease, the level of ceramide C24:1 and most of the complex glycosphingolipids was reduced. This may be explained by a decrease in de novo ceramide synthesis: mRNA levels of subunits 1, 2, and 3 of the long chain SPT and FVT1 were indeed significantly reduced, and the deregulation of its formation from hexosylceramides was also observed. GBA1 and GBA2 and GALC activities were reduced at the symptomatic stages of ALS development in SOD1^G93A^ mice. In the article by Shupik et al., a decrease in the total amount of ceramides in the spinal cord of FUS (1-359) line mice has been observed at the presymptomatic stage of the disease [94]. Decreased ceramide levels in the early stages of ALS can either occur during the development of the disease or be the manifestation of a compensatory response aimed at preventing the synthesis of toxic amounts of ceramide [81]. A work by Fernández-Beltrán et al. has indeed clearly illustrated the dependence of the levels of expression of sphingolipid metabolism enzymes from the stage of ALS [95].

### 4.2. Sphingoid Bases

Sphingoid bases (sphingosine and sphinganine) have bright proapoptotic properties. Their proapoptotic action is associated with the ability of these sphingolipids to interact with DNA, affect the activity of replication and transcription enzymes [99], and the DNA-binding properties of a number of regulatory proteins, including transcription factors and topoisomerases [100,101]. In contrast, sphingosine-1-phosphate has anti-apoptotic properties and proliferative effects.

Noteworthy in the context of ALS may be the ability of sphingosine-1-phosphate to inhibit histone deacetylase. Inhibition of histone deacetylases has recently been shown to regulate metabolic changes, including proper lipid homeostasis in FUS ^+/+^ spinal mice, as well as an ALS model with beneficial effects on motor phenotype and survival [102]. A special role in these processes is played by the enzyme sphingosine kinase, which reduces the level of sphingosine in the cell, thereby saving it from death [103,104]. Possessing bright proapoptotic properties, sphingosine and sphinganine can be directly involved in the death of CNS cells during the development of ALS. However, the involvement of sphingoid bases in motor neuron death is still virtually unknown. Integrated analysis of RNA-sequencing and lipidomic data from the spinal cord of symptomatic SOD1^G86R^ mice have revealed a correlation between disease severity, the content of sphingosine, and gene expression for its metabolism [105]. This work is the first one demonstrating a pronounced dysregulation in the metabolism of sphingoid bases, including sphingosine and sphinganine, in the FUS (1-359) transgenic mice model of ALS [96]. A significant increase in proapoptotic sphingosine and sphinganine has been found mainly in the spinal cord of mice, while the content of these sphingolipids was at a low level and remained virtually unchanged in brain structures during the development of ALS. This may be determined by the defeat of motor neurons in the disease. At the same time, the ratio of the anti-apoptotic agent sphingosine-1-phosphate to the pro-apoptotic sphingosine and sphinganine significantly decreased, which indicates cell death intensification in the structures of the spinal cord. Significant disturbances in the expression of sphingolipid metabolism genes at different stages of ALS development, mainly in the spinal cord, have been found. Of the four studied genes encoding ceramidase, the level of mRNA of acid ceramidase Asah1, localized in lysosomes, was the one increasing during the progression of FUS-mediated proteinopathy.

**Figure 2:**
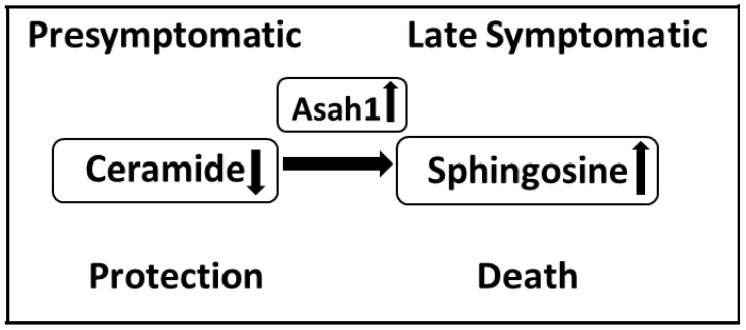
Changes in the sphingolipid balance in the spinal cord of FUS (1-359) mice during the development of ALS. The symptomatic stage is characterized by an increase in the content of ceramides and an increase in the expression of acid ceramidase, which leads to the formation of the proapoptotic agent sphingosine. A decrease in the content of ceramides at the presymptomatic stage may indicate a protective response of the sphingolipid system, activated by the body at the earliest stages of ALS.

Thus, given that sphingosine is generated from ceramides by lysosomal ceramidase, a change in its activity may indicate the development of “lysosomal” apoptosis. A jump in the sphingosine-1-phosphate lyase gene expression at the terminal stage of the disease further demonstrates a drop in the anti-apoptotic reserves of motor neuron cells and a rapid development of apoptosis at the terminal stage of the disease [96]. In Figure 2, based on a review of the literature and data obtained in our laboratory, we present possible stages characterising principle changes in the key pro-apoptotic components of the sphingomyelin cycle, ceramide, and sphingosine during the development of ALS in the spinal cord.

### 4.3. Lactosylceramides and Galactosylceramides

The formation of lactosylceramide C18:0 is observed in the spinal cord at the terminal stage of the disease in patients with ALS. This occurs against the background of increased activity of α-galactosidases (enzymes for the synthesis of lactosylceramide), both at acidic and neutral pH values [81]. The formation of lactosylceramide can contribute to the development of the disease, since it is a mediator of inflammation and apoptosis [106] and activates microglia via the NF-κB signaling pathway [107], which is involved in the death of motor neurons in ALS [108]. The content of the C24:1 form of galactosylceramide is increased in the white matter of the cervical spinal cord in patients with sporadic ALS [81]. An excess of galactosylceramide in cells can also negatively influence the course of the disease. For example, impaired metabolism of galactosylceramide due to mutations in GALC is involved in Krabbe disease, a pathology characterized by severe motor impairment [109]. The formation of ceramide in spinal cords of transgenic mice FUS (1-359) models of ALS at the symptomatic stage may be related to increased gene expression of the Galc enzyme, which generates ceramide from galactosylceramide. These are all features that we have previously observed to be associated with increased expression of the Galc gene. Conversely, the reverse process, namely, the formation of galactosylceramide from ceramide, is inhibited, since the expression of the Ugt8a gene is manifested [94].

### 4.4. Glucosylceramides

The analysis of homogenates of the cervical post-mortem spinal cord of ALS patients has shown an increased content of glucosylceramides C18:0 and C24:1. The analysis of spinal cord samples from mice of the SOD1^G93A^ line, isolated for different periods of the disease, has also shown an increase in the content of the glucosylceramide C24:1 metabolite at the terminal stage of ALS [81]. When studying mice of the SOD1^G86R^ line, A. Henriques et al. [92] found significant changes in the composition of sphingolipids already at the presymptomatic stage of ALS, and not only in the CNS, but also in the muscles of transgenic animals. While the levels of most of the glucosylceramides studied by the authors were reduced in the spinal cord of presymptomatic and symptomatic mice, the levels of many of these glucosylceramides were increased in muscles at the same stages of ALS. The analysis of spinal cord samples has also shown an age-related decrease in total glucosylceramides in both SOD1^G86R^ and control mice. In muscle tissues, age-related changes in the level of glucosylceramides have not been observed [92]. The content of glucosylceramides and, accordingly, the first stage of the biosynthesis of complex glycosphingolipids, seems to be controlled by glucosylceramide synthase (GCS), a transmembrane protein of the Golgi complex [110]. The level of GCS mRNA expression has been found to be significantly increased in the muscles at the asymptomatic and symptomatic stages of ALS in SOD1^G86R^ mice.. An increase in the level of GCS mRNA in the early stages has been considered by the authors as an event preceding the onset of a manifest motor disease. Western blotting and muscle immunohistochemical analysis also showed an increase in GCS protein, consistent with the observations described above. It is important to note that, in ALS, the GCS protein colocalizes with TDP-43, the main component of protein inclusions characterizing ALS pathology in humans [92]. Experiments using GCS inhibitors have supported the hypothesis of a protective role for glucosylceramides in ALS. The addition of the GCS inhibitor GENZ-667161 to SOD1^G93A^ mice has been observed to worsen the course of the disease, significantly accelerating the onset of paralysis and reducing the life span in animals [81]. Intraperitoneal administration of the GCS inhibitor AMP-DNM to SOD1^G86R^ mice has been found to lower the concentration of glucosylceramides and block the expression of a number of genes critical for the development of oxidative processes accompanying denervation. Thus, AMP-DNM significantly reduces the expression level of PGC1α, the main regulator of mitochondrial biogenesis, as well as PPARα, an activator of lipid catabolism, as well as lipoprotein lipase, which hydrolyzes triglycerides. In addition, inhibition of GCS has been found to slow down the recovery of motor functions, which suggests a critical role of GCS in response to muscle degeneration [92]. An increase in GCS activity (i.e., intensification of the formation of glucosylceramide from ceramide) has been identified as a negative regulator of ceramide-induced apoptosis [111], which once again indicates a potential protective role of glucosylceramide in ALS, possibly through the weakening of the toxic effect of ceramide. Inhibition of GBA2, an enzyme forming ceramide from glucosylceramide, is expected to have the opposite effect of GCS inhibition. Conduritol B epoxide, a GBA2 inhibitor, has been found to increase the level of glucosylceramide, significantly attenuating the deregulation of genes involved in ALS pathogenesis, and they also preserve the functionality of the neuromuscular junction, preventing motor neuron death in a SOD1^G86R^ mice model of ALS [112]. In addition, the intensification of the formation of glucosylceramide from ceramide is thought to have a beneficial effect on the course of the disease and slow down the development of ALS, initiating the synthesis of complex neurotrophic ganglioside sphingolipids, which promote neuronal growth and axonal development [113].

### 4.5. Gangliosides

Indications of deviations in ganglioside homeostasis, a glycosphingolipid with one or more sialic acids, in ALS, appeared in the literature at the end of the last century. Unique gangliosides [114], antibodies to gangliosides GM2 and GM1 [115,116], and an increase in the level of GM2 in the motor cortex of the brain of ALS patients [117], have been subsequently found. Recent studies have shown that the total content of the neurotrophic ganglioside GM3, as well as the content of its molecular species C18:0 and C24:1, is significantly increased at different stages of ALS in SOD1^G93A^ model mice [80]. This can slow the progression of the disease by stimulating oligodendrocyte differentiation, which is impaired in ALS [118,119]. At the symptomatic stage of ALS in the spinal cord of SOD1^G86R^ mice, a significant increase in the content of GM1a, the main CNS ganglioside, has been observed, and muscles have been found to show a significant increase in the content of GM3 and GM2 gangliosides [92]. Gene expression analysis of SOD1^G93A^ mice has shown that the amount of hexosaminidase (HEX) mRNA, an enzyme that metabolizes GM2 to GM3, is increased in motor neurons of the spinal cord in both the asymptomatic and symptomatic stages of the disease [120,121]. Additionally, HEX activity is increased in the spinal cord of SOD1^G93A^ mice and patients with sporadic ALS [122], but this increase in HEX activity has not been found to ameliorate the course of the disease, a phenomenon possibly explained by a lack of endogenous substrate (GM2) for HEX. In accordance with this assumption, intraventricular infusion of exogenous GM3 to SOD1^G93A^ mice has been found to delay the onset of paralysis and increase the lifespan of animals [81].

### 4.6. Cholesterol and Its Relationship with Sphingolipids in ALS

Cholesterol plays an important role in membrane structure, particularly of brain cells, which contain up to 25% of all cholesterol. Cholesterol is mostly concentrated in the myelin sheath (up to 70% of total brain content), where its main function is to stabilize the membrane and increase its stiffness, which in turn contributes to the conduction of the electrical impulse. Much less cholesterol is contained in the plasma membranes of neurons (up to 10%) and glia (up to 20%) [123]. Neurons are characterized by a high concentration of cholesterol in synaptic membranes, which promotes neurotransmission and stabilization of cell contact. Notably, cholesterol synthesis in the adult brain is performed mostly in glial cells, not in neurons. Cholesterol is synthesized in astrocytes and forms a lipoprotein particle with ApoE, which is secreted into the external environment via endosomes. The cholesterol complex with ApoE is captured by neuronal membrane receptors and is included in nerve cell metabolism [124].

In the cell membrane, cholesterol, in combination with sphingolipids, forms special microdomains–lipid rafts, which also include specific protein molecules (receptors, ion channels, enzymes). Lipid–protein complexes of lipid rafts are involved in the processes of membrane transport, signal transduction, and formation of synaptic vesicles in presynaptic nerve endings. Cholesterol plays both a structural role (it enhances the rigidity of the synaptic membrane and supports the formation of membrane curvature during vesicle formation) and a regulatory role, during exocytosis, in the process of neurotransmitter release during synaptic transmission [125]. Cholesterol and sphingolipids, which are associated with proteins, are included in the raft structure as lipid components. In rafts, the main component of sphingolipids is ceramide or sphingomyelin, which consist of a hydrophobic ceramide and a hydrophilic head of phosphorylcholine. The close interaction between the sterol ring of cholesterol and the ceramide sequence of sphingomyelin provides a lateral interaction between sphingomyelin and cholesterol, forming a specific domain. In these microdomains, cholesterol performs a stabilizing function by filling the voids between the large volume of sphingolipids. The cholesterol–sphingomyelin interaction determines the transition of these domains into a liquid-ordered or jelly-like phase, which is a unique characteristic of rafts. At the moment, multiple cross pathways of cholesterol and sphingolipid biosynthesis have been studied both at the genetic level and in terms of their structural interaction in the composition of rafts. The content of cholesterol in the membrane clearly correlates with the content of sphingolipids [126,127]. A decrease in the levels of sphingomyelin and ceramide is accompanied by a reduction in the cholesterol content. It is possible that the primary cause of cholesterol accumulation may be an increase in sphingomyelin levels. It has been established that the catabolism of sphingolipids is associated with the catabolism of cholesterol. However, while the exact mechanism of this interaction is still unknown [128], it is well recognized that disturbances in one of them are clearly reflected in the catabolism of another lipid. For example, hydrolysis of sphingolipids affects cholesterol metabolism. Activation of sphingomyelinase, an enzyme that hydrolyzes sphingomyelin to ceramide, accelerates cholesterol esterification without increasing the pool of cellular cholesterol [129]. At the same time, sphingosine, which is a product of enzymatic degradation of ceramide, inhibits cholesterol esterification [130]. The close relationship between the metabolic pathways of cholesterol and sphingomyelin has also been confirmed in ALS pathology: in works in patients and mutant animal models, an increased content of cholesterol esters has been noted along with an increase in sphingomyelin and its metabolite ceramide in the spinal cord [91]. It has been shown that inhibition of sphingolipid synthesis prevents not only the accumulation of sphingomyelin, but also cholesterol esters [91].

The significance of cholesterol and its metabolites in various CNS pathologies has been widely studied. However, the role of cholesterol in ALS cannot yet be considered solved. Only few works focused on the role of membrane cholesterol in ALS mechanisms have been published. There is increasing evidence of a positive correlation between elevated plasma cholesterol content and survival in ALS [60]. In a large study of 512 ALS patients, hypercholesterolemia (elevated cholesterol content) has been observed in 73% of cases, and the study further demonstrated a statistically significant positive effect on the survival rate [55]. Additionally, high plasma cholesterol content against the back-ground of elevated triglycerides content has been found to exert a significant effect on patients’ survival [57]. However, in dyslipidemia, no correlations have been observed between ALS development and tri-glyceride and HDL-associated cholesterol content, while it has been shown that the content of total cholesterol and HDL cholesterol clearly correlate with the risk of ALS [15]. Probably, the observed differences depend on the nature of the disease course: in hyperlipidemia, high triglyceride and cholesterol content might be a positive factor; in dyslipidemia, this phenomenon may represent a risk factor. Correction of sphingolipid metabolism might therefore lead to a normalization of the cholesterol level in neurodegenerative diseases, including ALS.

### 4.7. The Potential of Fingolimod as a Therapeutic Strategy in ALS

A typical feature of ALS found in the nervous system and peripheral body fluids is neuroinflammation [131]. Although the development of ALS is the result of a slow and progressive dysfunction and loss of motor neurons, non-neuronal cells of the central and peripheral nervous system, including immune ones, play an important role in the development of the disease [132,133]. Spread and activation of microglia and astroglia are characteristic histological features of the spinal cord and motor cortex of ALS patients and can be detected in vivo by positron emission tomography [134,135]. In mouse models of ALS, it has been shown that microglial activation and T-lymphocyte infiltration into the CNS occur at the early stages of the disease or even before the onset of symptoms, and the severity of inflammation correlates with the development of ALS symptoms [132,136].

In ALS pathogenesis, two stages can be conventionally distinguished: an early neuroprotective one (the microglia is characterized by the M2 phenotype, and T-lymphocytes are represented by the Th2 and Tregs populations) and a late neurotoxic one (the microglia is characterized by the M1 phenotype, and T-lymphocytes are represented by the Th1 and Th17 populations) [137]. Attempts to use broad-spectrum immunosuppressants for the treatment of ALS have not been proven to be effective [50,138,139]. This may be due to the fact that the studied agents suppress both protective and cytotoxic cell populations [140]. A more targeted effect on the immune system has been achieved using a structural analogue of sphingosine, the drug fingolimod, which penetrates the BBB [141]. The biological activity of sphingosine was first shown in 1986, when Y. Hannun et al. found that sphingosine inhibits protein kinase C [142], and subsequent works have shown that sphingosine is also involved in cell cycle arrest and apoptosis by modulating protein kinases and other signaling pathways [143].

Endogenous sphingosine kinases phosphorylate fingolimod, which leads to the formation of fingolimod-phosphate, an analogue of sphingosine-1-phosphate with anti-apoptotic properties, by binding S1PR receptors [144], effectively modulating their level on the cell surface. Unlike traditional immunosuppressive drugs, fingolimod does not suppress the activity of T- and B-lymphocytes [145,146], but it reduces the migration of pathogenic lymphocytes into the CNS. Fingolimod also increases the number of tregs circulating in the blood, causing redistribution, rather than depletion, of lymphocytes [147]. The current therapeutic use of fingolimod in ALS is based on its immunomodulatory activity, although fingolimod is potentially able to induce a gene expression program in neurons that modifies the phenotype and may reduce the loss of neuronal connections observed in the course of neurodegenerative disorders [148]. The drug improves the neurological phenotype and increases the lifespan of SOD^1G93A^ mice with symptoms of ALS. The beneficial effect of fingolimod is associated with the modulation of neuroinflammatory and protective genes (*CD11b, Foxp3, iNOS, IL-1β, IL-10, Arg1, Bdnf*) in the motor cortex and spinal cord of animals. The analysis of the expression of genes associated with either the M1 phenotype (*iNOS, IL-1β*) or the M2 phenotype (*Arg-1, IL-10*) has revealed increased expression of the anti-inflammatory markers Arg1 and IL-10 and the neurotrophic factor BDNF, as well as a concomitant decrease in expression of iNOS and IL-1β in mice receiving fingolimod. In addition, fingolimod has been found to cause an increase in the expression of the transcription factor FoxP3 [136], an indicator of an increase in the population of treg cells [149]. Furthermore, fingolimod has been shown to significantly reduce CD11b mRNA expression in the lumbar spinal cord and motor cortex, while no such changes have been observed in the cervical spinal cord, indicating areas of selective suppression of microglia activation by the drug. It should be noted that the efficacy of fingolimod therapy in SOD1^G93A^ mice has been proven at the symptomatic stage of ALS, with diagnosed motor dysfunction, suggesting the potential of fingolimod as an extremely important approach to the treatment of sporadic ALS [136]. Currently, fingolimod is the only drug of a sphingolipid nature undergoing phase II clinical trials for the treatment of ALS [140]. However, in terms of drug effects on the sphingomyelin cycle in ALS, genetic inhibition of enzymes is another important area of focus. For example, in a recent study by Choi et al., genetic inhibition of acid sphingomyelinase, which degrades sphingomyelin to ceramide, improved motor impairment and loss of spinal neurons in the FUS-R521C mouse model of ALS, suggesting a role of acid sphingomyelinase as a potentially effective target, and its inhibition was considered a possible therapeutic approach for ALS [150].

## 5. Conclusions

The review has summarized the most popular models used to study ALS mechanisms. Among them, greater emphasis has been placed on ALS models using various rodent species, including mice and rats expressing mutant SOD1 isoforms. Mouse models have also been developed, expressing multiple copies of the mouse SOD1 mutant with early fatal motor neuron disease. In addition to murine models, transgenic rat strains overexpressing SOD1 have been developed, which have been proven to be particularly useful for evaluating therapeutic trials due to animal size advantages, especially when administering therapeutics, such as continuous intraspinal delivery of therapeutics. Due to the fact that, in most ALS patients, the pathology is caused by disturbances in RNA metabolism, multiple models have been created with mutations in proteins that ensure RNA metabolism, such as TDP-43 and FUS. The FUS and TDP-43 proteins have a similar domain structure and perform similar functions in the cell: they are involved in the regulation of mRNA processing and transport [81,83,87,88].

Numerous studies presented in this review, both in various ALS models and in patients, demonstrate a pronounced dysregulation in the metabolism of various lipid classes. Significant disturbances in the content and synthesis of lipids have been indeed identified at different stages of ALS. The composition of fatty acids in total lipids in the blood of ALS patients may reflect the underlying pathological condition. Based on changes in the content of some fatty acids observed during the development of ALS, namely, palmitoleate (16:1) and oleate (18:1), which have been found to correlate with clinical measures of disability (ALSFRS-R scale), it has been lately proposed to use the change in the ratio of these acids as an independent prognostic factor in the disease [59]. In addition to changes in the composition of fatty acids, differences in the content of phospholipids between ALS patients and controls, as well as between the transgenic ALS SOD1 ^G93A^ model and controls, have been reported [11].

Recently, special attention has been paid to the study of the role of sphingolipids in the development of ALS. Changes in sphingomyelins, ceramides, glucosylceramides, galactosylceramides, gangliosides, and others are being intensively studied in ALS animal models, as well as in patients. The use of mass spectrometry has enabled us to study specific differences between ALS patients and controls in the spectrum of molecular species of sphingolipids during the development of the disease. The results of these studies have been discussed in this review, and the mechanisms of sphingolipid metabolism disorders in ALS have been described. A deeper understanding of the biological pathways regulating the metabolism of various sphingolipids during the development of ALS may lead to the identification of new potential drug targets. In particular, enzymes of sphingolipid metabolism involved in the pathogenesis of ALS can act as such targets. Of particular interest as a potential new therapeutic strategy in ALS is fingolimod, a synthetic analog of sphingosine, which is phosphorylated in the body and has an effect characteristic of sphingosine-1-phosphate, an anti-apoptotic metabolite of the sphingomyelin cycle. Thus, future studies on changes in lipid metabolism in ALS have strong potential to provide a clearer understanding of the pathological mechanisms of ALS, as well as the development of new drugs for the treatment of this neurodegenerative disease.

## Figures and Tables

**Table 1:** Alterations of lipids in amyotrothic lateral sclerosis in different tissues (PC—phosphatidylcholine, SPM—sphingomyelin; nd—no significant differences patients and controls). Up arrow icon means that the content of the metabolite is increased, the down arrow icon means that it is reduced. Up and down arrows mean that date are inconsistent.

Lipid Type	Tissue	Alterations		References
Total lipids	Human plasma (ALS patients)	Dis- or hyper-metabolism (2/3 patiens-dislipidemia)	↓↑	[15,16,54]
Triglycerides	Human plasma (ALS patients)	elevated triglyceride had a longer life expectancy	↑	[54,57]
	Human cerebrospinal fluid (ALS patients)	long-chain triglycerides	↓	[11]
Phospholipids	Human cerebrospinal fluid (ALS patients)	PC (20:4), SPM (22:0) PC (36:4p), PC (36:4e)	↑	[11]
	Model mice brain	PC (36:2), PC (36:4), PC (40:6)	↑	[11]
	The spinal cord of transgenic SOD 1^G93A^ mice	PC (diacyl-16:0/22:6), PC (diacyl-18:0/22:6), PC (18:1/22:6)	↓	[58]
Polyunsaturated fatty acids	Human plasma (ALS patients)	Palmitoleate (16:1), oleate (18:1)	↑	[59]
Cholesterol	Human plasma (ALS patients)	Total cholesterol	↑ nd	[55,57,60] [54]

## Data Availability

Not applicable.

## Abbreviations

ALS	amyotrophic lateral sclerosis
ALSFRS-R	amyotrophic lateral sclerosis functional rating scale revised
ApoE	apolipoprotein E
Asah1	acid ceramidase
BBB	blood-brain barrier
BDNF	brain-derived neurotrophic factor
C9ORF72	chromosome 9 open reading frame 72
CNS	central nervous system
CSF	cerebrospinal fluid
FUS	fused in sarcoma
FVT1	3-ketodihydrosphingosine reductase
GCS	glucosylceramide synthase
HEX	hexosaminidase
HDL	high-density lipoproteins
IL-6	Interleukin-6
iPS cells	induced pluripotent stem cell
LPO	lipid peroxidation
LDL	low-density lipoproteins
MCT1	monocarboxylate transporter 1
Nrf2	nuclear factor erythroid-derived 2-like 2
PUFAs	saturated fatty acids
SMA	spinal muscular atrophy
SMS	sphingomyelin synthase
SOD1	superoxide dismutase 1
SPT	serine palmitoyltransferase
SPTLC1	subunit 1 of the long chain serine palmitoyltransferase
TDP-43	TAR DNA binding protein
TFIID	transcription factor II D
TNF-α	tumor necrosis factor
UGCG	UDP-glucose ceramide glycosyltransferase
UGT8	galactosyltransferase
VCP	valosin containing protein
